# Monkeypox Goes North: Ongoing Worldwide Monkeypox Infections in Humans

**DOI:** 10.3390/v14091874

**Published:** 2022-08-25

**Authors:** Barbara S. Schnierle

**Affiliations:** Section AIDS and Newly Emerging Pathogens, Department of Virology, Paul-Ehrlich-Institut, 63225 Langen, Germany; barbara.schnierle@pei.de

**Keywords:** monkeypox, vaccine, 2022 outbreak, orthopox

## Abstract

In the late 1970s, global vaccination programs resulted in the eradication of smallpox. The Monkeypox virus (MPXV), which is closely related to the smallpox-inducing variola virus, was previously endemic only in Sub-Saharan Africa but is currently spreading worldwide. Only older people who have been vaccinated against smallpox are expected to be sufficiently protected against poxviruses. Here I will summarize current knowledge about the virus, the disease caused by MPXV infections, and strategies to limit its spread.

## 1. The Poxvirus Family

Poxviruses are large DNA viruses that infect a wide range of hosts, including mammals, birds, reptiles, and insects. The poxvirus family is subdivided into two main families, the *Chordopox,* which infect vertebrates, and the Entomopox, which infect insects. *Chordopox* are further divided into eight genera. The Orthopox genus encompasses the human pathogens variola virus (VARV), the causative agent of smallpox, and the monkeypox virus (MPXV), as well as the cowpox virus, the camelpox virus, and the vaccinia virus (VACV), which has been used as a smallpox vaccine. All Orthopoxviridae generate cross-reactive humoral and cellular immune responses. Therefore, 200 years ago, Edward Jenner was able to use cowpox inoculations that do not cause disease in humans, as a smallpox vaccine [1]. This first successful vaccination campaign resulted in the eradication of VARV in the late 1970s.

Orthopoxviruses are large, enveloped, double-stranded DNA viruses with a genome of 180–220 kb encoding 180–200 genes. They replicate exclusively in the cytoplasm of the infected cell and consequently encode all enzymes needed for DNA replication and transcription. Virions are brick shaped and have a size of approximately 250 nm × 220 nm. Cell entry occurs through low-pH-dependent macropinocytic uptake, which releases the viral core into the cytoplasm [2]. In the viral core, early gene expression takes place and the virus uncoating is initiated [3]. This leads to DNA replication followed by intermediate and late gene expression. Progeny DNA molecules, enzymes packaged into virions, and structural proteins assemble to form the viral particles that undergo extensive maturation steps [4]. Poxviruses have two infectious mature forms: extracellular enveloped virus (EEV) and intracellular mature virus (IMV). It is believed that EEVs mediate the spread of the virus in an infected organism and IMV mediate host-to-host transmission [4,5,6]. 

## 2. Monkeypox (MPX)

After the eradication of VARV, MPXV became the most prevalent zoonotic orthopoxvirus infection in humans. MPXV was first identified in 1958 during an outbreak of a pox-like disease in macaque monkeys in a research institute in Denmark [7]. Later, in 1970, human cases were discovered in the Democratic Republic of Congo (DRC) [8]. MPXV infections that occur mainly in children were, until recently, only sporadically identified in DRC and other Central and West African countries where MPXV is endemic. The first MPX case outside Africa was a zoonotic transmission from a pet prairie dog to humans in the USA [9,10]. While VARV persisted only in primates including humans, MPXV is more promiscuous and can infect many species. The natural reservoirs of MPXV described so far are African squirrels, rodents, and non-human primates [11,12,13]; however, the susceptibility of other animal species outside Africa still needs to be determined in more detail.

There are two distinct genetic clades of MPXV, the Central African or Congo Basin (CB) clade, now called clade I, and the West African (WA) clade, now clade II. Both clades show different case fatality rates, with 10.6% for clade I and 3.6% for clade II [14]. Genomic analysis of the two clades identified a 10 kbp deletion in the less virulent clade II MPXV [15].

## 3. The 2022 MPXV Outbreak

Currently, an unusual outbreak of MPXV of the less virulent clade II is occurring outside Africa. Starting in May 2022, independent cases of MPXV infections with local transmissions have been reported. The WHO (World Health Organization) had described 25,047 confirmed cases outside Africa by 2nd August 2022. Of these, 99% were males, with a median age of 36 years. When sexual orientation was reported, 98% were men who have sex with men (MSM) [16]. In July 2022, the WHO declared the global spread of MPXV a public health emergency of international concern (PHEIC). 

## 4. Orthopoxvirus Physical Stability and Transmission

Orthopoxviruses are present in lesion crusts and secretions of infected individuals and can remain infectious in this environment for extended periods of time [17]. For instance, VARV survived in crusts for several weeks in a temperature- and humidity-dependent manner: 3 weeks at 35 °C and high humidity, and up to 12 weeks at 26 °C and low humidity [18]. VARV could be reisolated from dried crusts obtained from smallpox patients after several years [19]. Even in aerosols, VACV could survived for 23 h, depending on the temperature and humidity [20]. A high stability in food and in the environment has also been demonstrated for VACV [21]. However, common disinfectants are effective and orthopoxviruses including VARV can be inactivated by 70% ethanol, 50% isopropanol, 0.1–2% sodium hypochlorite, or 1% formaldehyde within one minute or by heating at 65 °C for 15 min [21,22]. 

Orthopoxvirus physical stability accounts for the virus transmission routes. Transmission can occur by direct contact with the blood, bodily fluids, or cutaneous or mucosal lesions of infected animals or humans. In addition, a large number of respiratory droplets containing the virus or eating incompetently cooked meat and other animal products from infected animals are possible risk factors. Human-to-human transmission can also involve contaminated objects. Therefore, health workers, household members, and close skin-to-skin contacts of active cases are at greater risk. After the eradication of smallpox, the population’s immunity to orthopoxviruses is gradually declining and current MPX cases are mainly in younger people < 45 years of age. The high prevalence of MPXV infections in MSM in the 2022 outbreak raises the question of whether MPXV can be transmitted specifically through sexual transmission routes or whether close physical contact is the sole transmission route [16]. 

## 5. Clinical Disease Caused by MPXV Infections

**Figure 1:**
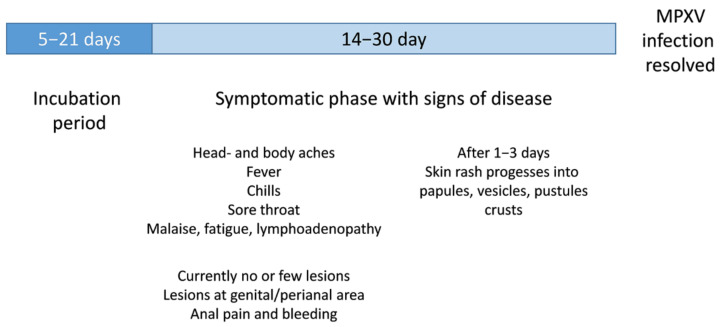
Schematic illustration of clinical signs of a MPXV infection.

The incubation period, the time from infection with MPXV to onset of signs of disease (MPX) normally ranges from 5–13 days, but can take up to 21 days. The first signs of disease are fever, intense headache, lymphadenopathy, sore throat, nasal congestion, cough, myalgia, and fatigue (Figure 1). Within 1–3 days of the appearance of fever, a rash appears on the face and extremities, rather than on the trunk. Oral mucous membranes, genitalia, and conjunctivae, as well as the cornea, are also affected, which can result in loss of vision. The rash evolves sequentially to papules, vesicles, pustules, and finally crusts, which dry up and fall off. The number of lesions varies from a few to several thousand. In the current outbreak, most cases have presented with mild disease symptoms and rash is the most frequently reported sign of disease and less lesions were reported [23]. Currently, rash and lesions appear mainly near the genitals or anus but also on other areas such as the hands, feet, chest, or face. The disease is usually self-limiting, but severe cases can occur in children, pregnant women, and immune suppressed people [16,24].

The case fatality rate of MPXV infections depends on health care system access and is lower in non-endemic regions. For the currently circulating clade II, the MPXV case fatality rate has been determined to be 3.6%. Among the 25,047 infections presently confirmed, 5 deaths have been reported in African regions and 2 outside Africa (2 August 2022).

## 6. Diagnostics

The disease symptoms seen in MPX are common to many other diseases, such as chickenpox and measles. The level of poxvirus in blood and body fluids is rather low, but skin lesion material contains enough MPXV to be used for PCR testing [25]. In addition, detection of MPXV-specific IgM indicates a recent infection; however, recent vaccination interferes with serological testing [16].

## 7. Treatment of MPXV Infections

Vaccination is considered to be the best preventive measure against orthopoxvirus infections. Orthopoxvirus infections generate humoral and cellular immune responses that are cross-protective towards other viruses of this genus. Therefore, smallpox vaccines are regarded to be sufficient to control MPXV outbreaks and vaccination is likely to be 85% effective [26]. However, data on real world effectiveness are currently not available.

First generation smallpox vaccines were efficacious and their application eradicated VARV infections, but they no longer meet current safety and manufacturing standards. Two next-generation vaccines that protect against smallpox and MPX are licensed in Europe and North America, and one in Japan. Until recently, there was no demand for these vaccines, so their supply is presently limited [27]. Nevertheless, due to the small number of infections, mass vaccination is not required nor recommended at this time for the current outbreak outside Africa [16].

The smallpox vaccine ACAM2000 licensed in North America is a replication-competent live VACV. It is derived from a clone of a first generation vaccine, Dryvax, and is now purified and produced using modern cell culture technology [28]. ACAM2000 is applied as a single dose by scarification with a bifurcated needle. However, as it is a replicating virus, it cannot be used in immunocompromised people [29]. During the MPXV outbreak in the United States in 2003, ACAM2000 was demonstrated to reduce the symptoms of MPX [30]. 

LC16m8 is a third-generation vaccine. It is an attenuated but still replicating smallpox vaccine derived from the Lister strain of VACV and has an improved safety profile. It is currently only licensed in Japan [31]. VACV Lister was passaged in primary rabbit kidney cells at low temperature (30 °C) for 36 generations to obtain the LC16m8 strain, which has lower neurotoxicity, but is still immunogenic [31]. LC16m8 has been shown to be protective against MPXV in animal models, but has the same exclusion criteria as ACAM2000 [32].

A fourth-generation vaccine is a replication-deficient, attenuated, smallpox vaccine based on the modified vaccinia virus Ankara (MVA). It is named IMVANEX (Europe, UK), IMVAMUNE (Canada), or JYNNEOS (USA), depending on the country the vaccine is licensed in. It has been approved by the Food and Drug Administration (FDA) and recently by the European Medicine Agency (EMA) for the prevention of MPX in adults aged 18 years or older. IMVANEX has to be administered via intra-muscular injection, as a prime/boost vaccination with two doses administered 4 weeks apart. As a replication-deficient virus, it can be used in patients with atopic dermatitis and immunodeficiency, and has shown protection against MPXV infections in animal models [33,34].

Smallpox vaccines induced a long-term protection against VARV and, although the data are not yet available, it is expected that this will also apply to MPXV. Ring vaccinations of close contacts of MPXV-infected individuals and probable post-exposure prophylaxis (PEP), ideally within four days of first exposure to prevent onset of disease, in addition to preventive vaccination of risk groups will serve as immediate measures to contain the current outbreak.

Passive immunization with vaccinia immune globulin (VIG) isolated from pooled blood samples of smallpox-vaccinated individuals can be envisioned as an intravenous application. VIG supplies are currently very limited, because it was developed as a treatment for severe smallpox vaccine-induced adverse events. However, it has been shown to be effective in MPXV infections [35].

Two oral drugs, Brincidofovir and Tecovirimat (ST-246), have been approved for the treatment of smallpox and have demonstrated efficacy against monkeypox in animals [36,37]. Tecovirimat interferes with the formation of EEV and thereby virus spread, and is FDA and EMA approved for emergency use [38]. Brincidofovir is a viral DNA polymerase inhibitor effective against orthopoxviruses and is approved in the USA [39]. 

## 8. Genomic Changes in the 2022 MPXV Genomes

MPXV isolates from the current outbreak are from a single clade and most likely have a single origin, very similar to viruses previously detected in Nigeria, the UK, Singapore, and Israel in 2017–2018 [24,40]. As DNA viruses, orthopoxviruses have much lower mutation rates compared to RNA viruses, because their viral DNA polymerase has a 3′–5′ proofreading exonuclease activity [41]. The central region of orthopoxvirus genomes is highly conserved and encodes essential genes that are required for virus replication. In contrast, the two terminal areas are hypervariable and may contain deletions and sequence rearrangements. These variable terminal regions contain the majority of the virulence and host-range genes [42]. Gene duplications and gene deletions by recombination enable double-stranded DNA viruses to adapt to environmental pressures, including host changes [43]. However, MPXV genome sequences currently show no large reductions in genome size [44].

Poxvirus genomes are unusually A/T-rich, which is an indication of non-random mutations. APOBEC3 (apolipoprotein B mRNA-editing catalytic polypeptide-like 3) enzymes can increase these mutation signatures. APOBEC3s are upregulated upon poxvirus infection and APOBEC3-G, -F, and -H are located in the cell cytoplasm, where poxvirus replication takes place. Although APOBEC3G does not affect VACV replication directly, it might increase the hypermutation rate and the likelihood of producing virus variants with altered characteristics [45]. Signs of microevolution have been observed in phylogenomic analyses of the 2022 MPXV genomes, indicating a potential adaptation of MPXV to humans [40]. The 2022 MPXV has been circulating since 2017 [40], but diverges on average by 50 SNPs (single-nucleotide polymorphisms), showing a mutational bias indicating APOBEC3 action. The number of SNPs is more than expected from previous substitution rates of orthopoxviruses [40]. Several point mutations in the 2022 MPXV genome have been described recently, but their functional impact on virus spread or human-to-human transmission is still unknown [46,47,48,49,50,51].

## 9. Outlook

The rapid spread of MPXV outside endemic regions bears some risk to global public health if it is not contained quickly. The main drivers for the present MPXV global spread include the cessation of smallpox vaccination in 1980 making younger people vulnerable to MPXV infections, the failure to restrain the spread of MPX cases in endemic regions, and an increased likelihood of exportation of the virus to other countries due to globalization and air traffic. Therefore, disease surveillance in endemic and non-endemic regions is essential to control further spread. At this time, it is not clear if the 2022 MPXV differs in host change, transmissibility, or pathology compared to previous isolates. This needs to be urgently established.

Although formal proof of clinical vaccine efficacy is lacking, smallpox vaccines are expected to induce a long-lasting immunity against MPXV. The 2022 MPXV infections have been almost exclusively concentrated in the MSM community, most likely through close skin contacts. To contain the outbreak, vaccinations should to be offered to this community, health care workers, and close contacts of MPX patients. The infection is not limited to men, but can be transmitted to anybody by close physical contact. In addition, vaccination campaigns in endemic regions could be envisioned to save lives and eliminate the source of future outbreaks.

## Data Availability

Not applicable.
